# USABILITY OF THE SMARTWATCH AS A REMINDER AID FOR OLDER ADULTS WITH DIVERSE COGNITIVE ABILITIES

**DOI:** 10.1093/geroni/igad104.2209

**Published:** 2023-12-21

**Authors:** Edie Sanders, Walter Boot

**Affiliations:** Florida State University, Tallahassee, Florida, United States; Florida State University, Tallahassee, Florida, United States

## Abstract

Smartwatches have potential to provide support for prospective memory, the ability to remember and carry out an intention in the future, crucial for maintaining health, independence, and social connections. Little is known about how older adults, particularly those with cognitive impairment, might interact with smartwatches. This study aimed to understand 1) the types of challenges older adults with and without cognitive impairment experience when using a smartwatch, and 2) the potential for smartwatches to serve as reminder aids for this population. Participants were twenty-seven older adults (age 60+) with normal cognitive functioning or cognitive impairment due to mild cognitive impairment, traumatic brain injury, or stroke. Participants were asked to use a smartwatch’s reminder notifications to remember to complete a daily survey for 10 days and completed demographic, neuropsychological, technology acceptance, and usability measures. Half were first asked to use their usual memory strategies without the smartwatch to remember to complete the daily survey for 10 days. Participants gave relatively low usability ratings for the smartwatches overall and reported a variety of challenges. The smartwatches did not appear to significantly help or hurt participants’ abilities to remember to complete the daily survey. Perceived usefulness of the smartwatches was significantly associated with subjective memory, suggesting that perceived memory challenges may play an important role in the adoption of smartwatches. Results suggest that the smartwatches used in this study may not be the best fit for supporting prospective memory and can inform the development of future efficacy tests and interventions involving smartwatches.

